# Disclosure without accountability: Air pollution reporting by major South African industrial companies

**DOI:** 10.5334/aogh.5437

**Published:** 2026-09-18

**Authors:** Lorren Haywood, Phyllis Kalele, Nomfundo Mahlangeni, Thandi Kapwata, Candice Nkambule, Tracey Laban, Caradee Y Wright

**Affiliations:** 1 Smart Places, Council for Scientific and Industrial Research (CSIR), Pretoria, South Africa; 2 Environment and Health Research Unit, South African Medical Research Council (SAMRC), Pretoria, South Africa; 3 Environment and Health Research Unit, South African Medical Research Council (SAMRC), Cape Town, South Africa; 4 Atmospheric Chemistry Research Group, Chemical Resource Beneficiation, North-West University, Potchefstroom, South Africa; 5 Department of Geography, Geoinformatics and Meteorology, University of Pretoria, Pretoria, South Africa

**Keywords:** Air pollution, ESG disclosure, environmental health, Johannesburg Stock Exchange, particulate matter, sulphur oxides, nitrogen oxides, sustainability reporting, South Africa, volatile organic compounds

South Africa’s industrial sector emits some of the highest concentrations of sulphur dioxide on the African continent, driven by a coal-dependent energy system and a resource-intensive industrial base concentrated in regions such as the Highveld and Vaal Triangle Airshed Priority Areas [1, 2]. Communities in these areas bear a disproportionate health burden, with chronic exceedances of National Ambient Air Quality Standards linked to elevated rates of respiratory morbidity and premature mortality [3, 4]. Against this backdrop, a critical question arises: does the growing corporate apparatus of Environmental, Social, and Governance (ESG) reporting actually translate into cleaner air?

The evidence is not encouraging. Our recent content analysis of 23 energy-intensive and high-emitting companies listed among the top 100 on the Johannesburg Stock Exchange (JSE), drawn from mining, metals, chemicals, industrial materials, and construction sectors, examined air pollutant disclosures across their 2022–2024 reporting period against the requirements of Global Reporting Initiative (GRI) Standard 305-7. This standard requires companies to report, in mass terms, their emissions of nitrogen oxides (NO_x_), sulphur oxides (SO_x_), particulate matter (PM), volatile organic compounds (VOCs), and other significant air pollutants, together with the methodology used to calculate them. The integrated annual reports and/or stand-alone sustainability/ESG reports were coded for each of the 23 companies by looking at reporting on disclosed (i.e., single number/set of numbers) and reduced (i.e., one number versus another number) emissions. The findings show that industrial companies have learned the language of ESG reporting without substantively changing what comes out of the smokestacks.

Most companies reported emissions data for SO_x_ and NO_x_, consistent with GRI 305-7 requirements. The gaps were, however, notable: four companies in the mining and metals sector did not disclose SO_x_ emissions, and five omitted NO_x_, despite these being signature pollutants from smelting and energy-intensive processing. For pollutants PM_2.5_ and PM_10_, VOCs and total suspended particulates (TSDs), disclosure was substantially less and inconsistently maintained across years (Note: Total Suspended Particulates were not coded as distinct from PM₂.₅ and PM₁₀ in the analysis). This mirrors global findings where a recent GRI analysis of 1000 organisations found that air pollutant reporting tends to be incomplete, uneven across pollutant types, and poorly correlated with actual environmental performance [5].

More troubling than what companies failed to report is what the reported data actually highlight. Among the 15 companies that provided quantitative emissions data, there was no consistent pattern of sustained year-on-year reduction. For SO_x_, 10 companies recorded emissions reductions in 2022–2023, but only eight recorded a reduction the following year. These findings were for the same set of companies suggesting intermittent improvements. NO_x_ trends were similar. PM disclosures increased over time but largely reflected expanded reporting coverage rather than emissions reductions. This highlights oscillation rather than progress: improvements in one year reversed the next, suggesting episodic rather than structurally embedded mitigation of air pollution.

This pattern is well explained by legitimacy theory, where stakeholder pressure compels companies to appear environmentally responsible rather than to become so. They publish disclosures that meet reporting norms without making the costly operational changes that actually reduce emissions [6–8]. Mandatory ESG disclosure by JSE-listed companies, introduced in 2010, was in part a response to voluntary reporting failures and persistent non-compliance with environmental standards [9]. Yet mandating disclosure, without mandating improvement, creates conditions in which reporting becomes an end in itself: a mechanism of corporate legitimacy rather than an instrument of environmental accountability.

South Africa’s regulatory architecture reinforces this dynamic. The National Environmental Management: Air Quality Act (Act 39 of 2004) establishes legally binding emission limits through the Minimum Emission Standards (MES), but enforcement has been challenging. Industrial operators have repeatedly secured postponements and exemptions from compliance, justified on grounds of technical feasibility or economic burden [10]. The result is a governance gap in which the formal strictness of the legislative framework is routinely softened in practice, removing the enforcement mechanism that would otherwise ensure sustained emissions reductions. Corporate sustainability reports can then present narratives of environmental stewardship without these being tested against enforceable performance benchmarks.

The consequences are not abstract. In communities surrounding the Highveld Priority Area, many of them already socio-economically marginalised, poor air quality is not a sustainability metric; it is a lived condition affecting respiratory and cardiovascular health, child development, and quality of life [1, 4]. Incomplete or inconsistent corporate disclosures constrain the ability of affected communities, civil society organisations, and regulators to assess environmental impacts, identify underperforming operators, and hold companies accountable. In this context, deficiencies in disclosure are not merely technical shortcomings; they are a form of information asymmetry with tangible public health consequences.

The selective character of what gets disclosed is also worth interrogating. The disproportionate focus on SO_x_ and NO_x_, both of which are greenhouse-gas-adjacent pollutants closely tracked by climate frameworks, relative to the underreporting of PM and VOCs reflects a broader distortion in ESG reporting: a tendency to prioritise globally visible climate metrics at the expense of locally salient air pollution-health impacts [5, 11]. Investors and regulators focused on decarbonisation may be inadvertently incentivising a form of disclosure that looks good in sustainability indices while leaving unaddressed the pollutants most damaging to the communities living adjacent to these operations.

What would more meaningful reporting look like? At minimum, it would require mandatory rather than voluntary disclosure of criteria air pollutants, including PM_2.5_, PM_10_, and VOCs, with standardised methodologies to ensure comparability across firms and years. Second, it would require an explicit link between disclosed emissions and regulatory compliance status, including any postponements or exemptions in force. Finally, it would require performance-based accountability rather than recognising disclosure as an endpoint; regulators and investors should treat it as a starting point for assessing whether operational emissions are actually declining.

Comparable regulatory shifts are already under way elsewhere. In the European Union, in-scope companies must now report pollutant releases to air on a mandatory, audited basis under the European Sustainability Reporting Standard on Pollution (ESRS E2) as part of the Corporate Sustainability Reporting Directive. Separately, the International Financial Reporting Standards (IFRS) Foundation’s climate-related disclosure standard, IFRS S2, while focused primarily on greenhouse gas emissions rather than criteria air pollutants, has already been adopted on a mandatory or voluntary basis in more than 20 jurisdictions, illustrating that standardised, enforceable sustainability disclosure of this kind is both feasible and increasingly the international norm. South Africa’s continued reliance on voluntary, GRI-referenced reporting, without a comparable compliance or assurance requirement, leaves it a notable outlier relative to this emerging regulatory trend.

There is growing appetite for precisely this kind of reform. The JSE’s Sustainability Disclosure Guidance acknowledges stakeholder demand for more harmonised, verifiable, and performance-oriented reporting [12]. The GRI itself has recently flagged the air pollution reporting gap as a priority concern [5]. In South Africa, where the scale of industrial air pollution and the regulatory enforcement gap are both well documented, the case for moving beyond voluntary reporting frameworks is compelling. Remote sensing and machine learning tools now offer promising supplementary approaches for detecting compliance deviations in real time, providing an independent check on self-reported corporate data [13].

ESG reporting was never designed to be an end in itself. If air quality is to improve in South Africa’s heavily industrialised regions and if the communities bearing the health burden of that pollution are to see genuine accountability, then the relationship between what companies disclose, what they actually emit and the corrective actions that they take needs to be far more rigorously examined, enforced, and, where necessary, mandated. Transparency is a necessary condition for accountability. It is not, on the evidence presented here, a sufficient one.

## Author Verification Statement

All authors had full access to the data used in this study and contributed substantially to the writing, review, and approval of the manuscript prior to submission.
